# Post-production Losses in Iodine Concentration of Salt Hamper the Control of Iodine Deficiency Disorders: A Case Study in Northern Ethiopia

**DOI:** 10.3329/jhpn.v28i3.5550

**Published:** 2010-06

**Authors:** Dawit Shawel, Seifu Hagos, Carl K. Lachat, Martin E. Kimanya, Patrick Kolsteren

**Affiliations:** ^1^ Department of Public Health, Mekelle University, Mekelle, Ethiopia; ^2^ Department of Food Safety and Food Quality, Faculty of Bioscience Engineering, Ghent University, Belgium; ^3^ Nutrition and Child Health Unit, Department of Public Health, Prince Leopold Institute of Tropical Medicine, Antwerp, Belgium; ^4^ Directorate of Food Safety, Tanzania Food and Drugs Authority, Dar es Salaam, Tanzania

**Keywords:** Cross-sectional studies, Iodine, Iodine deficiency, Salt, Ethiopia

## Abstract

Iodine is essential for good function of the thyroid, and its deficiency is of public-health importance in Ethiopia. Iodization of salt is an effective and sustainable strategy to prevent and control iodine deficiency in large populations. The effectiveness of salt-iodization programmes depends on the conservation of iodine concentration in salt at various stages of the supply-chain. The overall objective of the study was to assess the loss of iodine in salt from production to consumption and to estimate the proportion of adults, especially pregnant women, at risk of dietary iodine insufficiency. A cross-sectional study was conducted during February-April 2007 in northern Ethiopia. Iodine concentrations of salt samples from producers (n=41), retailers (n=7), and consumers (n=32) were determined using iodiometric titration. A risk assessment was conducted for dietary iodine insufficiency among adults, including pregnant women, using a semi-probabilistic approach. The concentration of iodine in the sampled salts decreased by 57% from the production site to the consumers. The assessment of exposure showed that adults in 63% (n=20) of the households, including 90% (n=29) with pregnant women, were at risk of insufficient iodine intake. A monitoring and evaluation system needs to be established to ensure adequate supply of iodine along the distribution chain. Special attention is needed for the retailers and consumers. At these levels, dissemination of information regarding proper storage and handling of iodized salt is necessary to address the reported loss of iodine from salt.

## INTRODUCTION

Iodine is an essential trace element for good function of the thyroid, which, in turn, is indispensable for optimal health. Deficiency in iodine is the single most common cause of preventable mental retardation and brain damage in the world. Iodine deficiency disorders manifest in decreased child-survival rates, goitre, and overall impaired growth and development (1–3).

The global database of the World Health Organization (WHO) shows that, during 1993–2003, iodine deficiency was still a public-health problem in 54 countries, and an estimated two billion individuals had an insufficient dietary iodine intake (4). In Ethiopia, around 28 million people suffer from goitre, and more than 35 million people are at risk of iodine deficiency. More importantly, 50,000 perinatal deaths are related to iodine deficiency each year in Ethiopia. The education potential of the nation is unattained as iodine deficiency may cause an intelligence quotient reduction of 13.5 points (5). The problem is both a threat to the productivity of the workforce and a cause of cretinism and mental retardation. These factors are estimated to lead to an enormous loss of gross domestic product. In Ethiopia, this has been estimated at nearly US$ 1 billion over the 2000–2005 period (6).

Iodization of salt is an effective and sustainable public-health strategy to prevent and control iodine deficiency and has been ongoing in several countries for over 60 years. Iodization of salt is currently undertaken following the universal salt-iodization initiative (7–9). Iodine is added to salt in the form of potassium iodide or iodate. At the point of production, it is recommended that the salt contains 20–40 mg of iodine per kg (10).

The actual availability of iodine from iodized salt at the consumer level can vary widely due to a number of factors: variability in the amount of iodine added during iodization, poor mixing resulting in uneven distribution within the batches or bags produced and instability of iodine in the salt. These factors affect how much iodine is finally available for consumption. Various factors, such as moisture content of salt, ambient humidity, light, heat, impurities in salt, alkalinity or acidity, and the form (potassium iodide or iodate) in which the iodine is present, affect iodine stability in salt (11). Hence, the effectiveness of salt-iodization programmes to deliver adequate amounts of iodine at the consumer level largely depends on the stability of iodine (10, 12–24).

According to the Ethiopia Demography and Health Survey 2005, only around 4.2% of the Ethiopian population uses iodized salt (25). Recently, a national iodization strategy was developed, and a 3–5-year supply of potassium iodate has been distributed to salt suppliers. To warrant adequate supplementation of iodine at the consumer level, monitoring and evaluating the concentration of iodine in salt (in particular by measuring iodine concentrations and documentation of losses) is an essential element of a programme to eliminate iodine deficiencies. Additionally, it is important to monitor what percentage of the population remains at risk of insufficient intake of iodine. Therefore, this study estimated the loss of iodine from production (iodization) to the consumption at the household level and performed an assessment of exposure to estimate the adequate coverage of iodine intake by adults and pregnant women in Tigray region northern Ethiopia.

## MATERIALS AND METHODS

### Study region

A cross-sectional study was conducted in Tigray region in northern Ethiopia from February to April 2007. The region is mountainous, with an average elevation of 1,972 metre above the sea-level. According to the Central Statistical Agency of Ethiopia, the region had a predominantly rural population of approximately 4,334,996 in 2005. The total goitre rate in the region was estimated to be 35.6%, and approximately 9.3% of the population reportedly knows the importance of iodized salt (26).

### Sampling of salt

The region is mainly served by one small-scale regional producer of iodized salt in Mekelle, the capital city of the region. Potassium iodate is used for iodization of salt. The iodized salt is packed by the producer in a 1-kg or a 50-kg polyethylene bag. According to the needs of consumers, this can be repacked in smaller plastic bags or paper packets. The iodization is performed on a weekly basis for about 4–5 hours a day. Four production days were randomly identified for sample collection at the production site. In total, 41 samples were collected at 30-minute intervals during the process of iodization over four days. The samples were taken immediately after salt had been iodized. Approximately 100 g of iodized salt was taken for each analysis. Samples were transferred to an airtight plastic cup and subsequently labelled and coded.

### Sampling from retailers and consumers

To determine the concentration of iodine in the iodized salt at the retailer and consumer levels, we followed the distribution of the iodized salt from the production site to the households. A town named Wukro, 50 km away from the production site in Mekelle city, was selected for the sampling. Wukro is the study site for the community health internship programme of the College of Health Science of the Mekelle University. A team of 15 undergraduate public-health students was trained as data collectors for the study. The producer sold salt to seven retailers in the study area. All retailers were included in the study. In total, seven samples were obtained from the retailers.

After obtaining informed consent from the retailers, a list of customers who were supplied with iodized salt for the past two months by the retailer was compiled. In total, 32 salt samples were collected from all the households based on this list. All the consumers on the list were traced, and salt samples were collected from their households.

A sample of approximately 100 g of iodized salt was taken from the retailers and consumers. The samples were transferred to an airtight plastic cup and subsequently labelled and coded. All the salt samples were transported to the Laboratory of Analytical Chemistry, Mekelle University, and were analyzed immediately after arrival. It took about two hours to transport the samples to the laboratory.

### Analysis of samples

To determine the concentration of iodine in the salt, trained laboratory technicians analyzed all the samples using iodiometric titration method approved by the World Health Organization/United Nations Children's Fund/International Council for the Control of Iodine Deficiency Disorders (WHO/UNICEF/ICCIDD) (10). Each sample was analyzed in triplicate. The average of these was taken as iodine concentration of the sample. The conversion of the titration results to iodine concentrations was done using a standardized table as per recommendations of the ICCIDD/MI/UNICEF/WHO (27).

### Exposure assessment

We carried out an assessment of exposure for adults and pregnant women who might consume iodized salt with the iodine concentrations at the production, retailer and household levels. We carried out the assessment of exposure for salt at the production level because the producer sells salt directly to consumers in a small shop located nearby the production site. The exposure assessment was conducted for the daily intake of iodine using a semi-probabilistic approach and by combining the average reported salt intake of 4.6±3.5 g per person per day reported by Abuye *et al.* for adults in Ethiopia with the intake of iodine (μg/day) from the different samples (28).

The WHO, UNICEF, and ICCIDD recommend that the daily intake of iodine should be 150 μg for adults aged over 12 years and 250 μg for pregnant and lactating women (10). The currently-used tolerable upper intake level for adults and pregnant women is 600 μg per day according to the European Commission's Scientific Committee for Food (EC/SCF) and 1,000 μg per day according to the Institute of Medicine (29).

As Thomson *et al.* did, we used a semi-probabilistic approach for the assessment of exposure with a fixed value for salt intake (30). This approach is appropriate when little variation in salt intake is expected. The diet of the households in the study area is monotonous, and as most households are poor and have a similar socioeconomic status, food intake consequently is expected not to vary much among different households. Hence, little variation in salt intake is anticipated.

We first performed the assessment of exposure using iodine concentrations from our samples. To evaluate the actual concentration of iodine in the diet consumed, we subsequently repeated this assessment taking into account an additional loss of 20% of iodine concentrations during the cooking process as estimated by the WHO (10).

### Analysis of data

Data were entered into MS Excel and were analyzed using the Stata software (Intercooled Stata version 10) (Statacorp, College Station, Texas, USA). In the case of severe departure from normality, data were transformed logarithmically until a normal distribution was obtained. All analyses were carried out with a significance level of 5%, and all tests were two-sided. Analysis of variance (ANOVA) test was used for exploring the differences of iodine concentrations in the salt between sampling levels and days of sampling.

### Ethical clearance

The Ethical Committee in the Mekelle University approved the study. Informed consent was obtained from the subjects involved in the study. The study subjects were informed about the objective of the study. The participants were allowed to consider their participation and given the opportunity to withdraw from the study at any point in the course of the study if they wished to do so.

## RESULTS

### Concentration of iodine in salt at production site

In total, 41 salt samples from the production site were analyzed. The concentration of iodine in salt found at the production site was 57.9±15.31 mg/kg. The mean iodine concentration was not different between the days of sampling (p=0.14). None of the samples had an iodine concentration below the minimum recommended iodine concentration of 20 mg/kg, and the maximum concentration found was 98.4 mg/kg.

### Iodine concentration in salt at retailers

The concentration of iodine in salt found at the retailers was 41.3±14.3 mg/kg. None of the samples had an iodine concentration of below 20 mg/kg. There was no difference in the mean concentration of iodine in salt from different retailers (p=0.45).

### Iodine concentration in salt at consumer level

The mean concentration of iodine in salt sampled from the study households was 25.1±22.5 mg/kg. The iodine concentration ranged from 1.1 mg/kg to 69.8 mg/kg in the samples.

The average decrease in iodine concentration of salt samples from the production to the retailer level was 29% and 50% from the retailer to the consumer level. On average, the iodine concentration was reduced by 57% from the production to the consumer level (Fig.).

**Fig.**
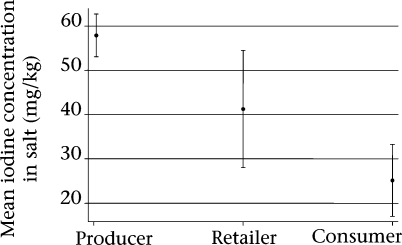
Iodine concentration (mg/kg) of iodized salt at production, retailer and consumer levels (mean and 95% confidence interval)

Place of sampling

### Assessment of exposure

Using the iodine concentrations as determined at the production, retailer and consumer levels, the daily intake of iodine was estimated to be 266 μg per person per day, 238 μg per person per day, and 119 μg per person per day respectively.

The table shows that 27% and 63% of the adults were exposed to the insufficient intake of iodine when consuming salt with iodine concentrations as observed at the retailer and household levels. Most (90%) pregnant women consuming salt with an iodine concentration observed at the household level were exposed to the insufficient intake of iodine. The tolerable upper intake levels of iodine did not exceed when consuming salt from the different sources. Taking into account an additional loss due to cooking, only a minority of the pregnant women was expected to have sufficient intakes of iodine when using salt from the retailers (7%, n=2) or salt observed at the household level (6%, n=2).

**Table:** Estimated iodine intake based on iodine concentration in salt from different sampling units at production, retailer and household levels

Place of sampling	No.	Mean iodine intake±SD	Range of iodine intake	Exposure assessment not adjusted for cooking losses		Exposure assessment adjusted for cooking losses*	
		(μg/day)	(μg/day)	Units <150 μg/day (%)‡	Units <250μg/day (%)‡	Units <150μg/day (%)‡	Units <250 μg/day(%)‡
Production	41	266.3±70.4	92.5–452.6	5	41	18	70
Retailer	7	189.0±65.8	136.2–311.4	27	82	49	93
Household	32	115.5±103.5	5.1–321.1	63	90	71	94

*WHO estimates that 20% of iodine is lost during cooking;

‡150 μg/g/day is the minimum requirement for iodine intake in an adult;

†250 μg/g/day is the minimum requirement for a pregnant woman;

SD=Standard deviation;

WHO=World Health Organization

## DISCUSSION

The study, for the first time, evaluated the losses of iodine occurring under local conditions between the time of iodization and the time of consumption in Ethiopia. This investigation is an independent study and not a monitoring and evaluation activity within the iodization programme.

Our findings show how the iodization and management of the supply-chain is currently not providing salt with sufficient iodine concentrations to the adults and pregnant women living in the target area. It is important to note that the decrease is more profound at the retailer and consumer levels. The losses are comparable with data documented from Nepal (31). In another study, Taga *et al.* measured large decreases in iodine concentration of salt at various points in the supply-chain and documented losses of around 44.8–82.3% (14). Similarly, different authors have described a high variability of iodine concentrations at the retailer or household levels (12, 14–16, 18–20, 24).

The findings presented here show that the losses of iodine are considerable. Only the concentrations at the production site were sufficient to ensure an adequate intake. The loss in iodine concentration at the household level is more than that estimated by the WHO—a reduction of 20% of iodine concentration from the production to the household level (10). The iodine concentration is anticipated to decrease further with additional losses during cooking. Using an additional 20% loss during cooking, it is apparent that a very few pregnant women are expected to consume sufficient iodine.

Contrary to previous findings (32), we did not observe co-existence of lower and higher iodine concentrations in salt in the households in the same community. The iodine concentrations in salt were overall low in the different samples.

One way to deliver sufficient iodine at the household level is to increase the concentration of iodine at the production site. This would, however, potentially expose consumers to excess intake levels. Taking into account the observed losses in the present study and those expected during cooking, a concentration of 119.2 mg/kg is needed at the production site to protect 50% of pregnant women. This concentration is double of what was measured in the study. When salt with this concentration is consumed, it would expose 31% of adults to intakes higher than the tolerable upper intake level of 600 μg per day according to the EC/SCF (29). Taking into account the losses observed after production, no consumers would be exposed to unacceptable high intake levels when this salt is consumed at the retailer or household level.

The observation of the magnitude of losses, however, and of the scarcity of published data from other iodization programmes is worrying and calls for additional studies to determine the actual concentration of iodine consumed at the household level.

There are several limitations to this study. We were unable to carry out an assessment of exposure for children as there was no average salt-intake estimate for this age-group. This limits the application of our findings to the whole population. Seasonal variations and changes in climatic conditions are expected to affect the concentration of iodine in salt. The findings of the study might, therefore, vary over the year, and our conclusions can only be related to the study season. In addition, the study area is located 50 km away from the production site, is semi-urban, and is relatively closer to the production site than many other outlet facilities in the region. Since losses of iodine are expected to increase with longer supply lines, the selection of this town might have underestimated the level of iodine loss.

Another limitation is the low sample size of the study. This may have affected our estimates on the variability of iodine in the samples and overestimated the probable effects to some extent. We are confident, however, that our point estimates are accurate and that our conclusions relating to the reduction of iodine in the samples are robust. Despite the small sample size, this study provides evidence to justify further research to study the population-wide effects of iodine losses in salt under field conditions.

We argue that the bias of the study area (a community study site) is minimal since this was the first time this area was selected for research with regard to iodine or salt.

Due to the cross-sectional nature of the study, we were unable to accurately determine the effect of time on the reduction of iodine concentration after production. There was no registration mechanism which allowed us to determine the exact production date and age of salt analyzed from the retailer and at the household level. The iodization date of salt was not recorded on the packages. Salt-bags from different production batches are mixed once they reach the retailer level. From the accounts of the retailers and households, we estimated that salt sold at the retailer level was iodized 2–3 months earlier. The salt sampled in the households originated from the same batch of production as the salt analyzed from the retailers and was stored an additional 1–2 months at home before analysis. We carried out an assessment of exposure assuming that iodine in the diet is negligible. As described earlier, the study area—Tigray region—is one of the highest affected areas by iodine deficiency in the country (26). We argue from this that the endogenous dietary supply of iodine is minimal and is not expected to affect the findings of our study.

The iodization of salt was done using potassium iodate, which is considered the most stable form for iodization in tropical climates (10, 11). The measurement of the moisture content, impurities, and acidity/alkalinity of salts at the production, retailer and household levels could have been helpful in explaining the significant loss of iodine concentration at the retailer and consumer levels.

Recommendations for iodine concentration of salt at the production level need to take into account local losses to ensure adequate iodine intake. Our findings call for the establishment of local standards for salt iodine levels. In addition, storing of salt in a dry place and a clean airtight plastic container and also avoiding excess exposure to sunlight and heat could possibly reduce the level of post-production loss of iodine at the retailer and consumer levels.

The findings clearly have important implications for the development of post-production quality-control and monitoring schemes, particularly during salt distribution and storage. A monitoring and evaluation system needs to be in place along the chain to ensure the adequate supply of iodine. This should also include printing of the production date on salt packets. Specific attention and collaboration with the retailers is necessary to address the loss of iodine at this level of the chain. Creation of awareness about proper storing and handling of iodized salt may prevent possible loss of iodine at the retailer and consumer levels.

## ACKNOWLEDGEMENTS

The authors gratefully acknowledge the financial support of the Mekelle University and NORAD II project. They thank Mr. Tekelit Gebregiorgies and Tame Kiros for their work during laboratory analysis. Special appreciation goes to Afework Mulugeta, Yemane Ashber, and Awala Equar, community health internship students, the salt-producing company, retailers, and consumers at Wukro district for their contribution to data collection of the study. The authors also thank Anne Mullen for the language revisions.
